# Experience of a Young Homœopath

**Published:** 1887-05

**Authors:** F. E. Stoaks

**Affiliations:** Attica, Ohio


### EXPERIENCE OF A YOUNG HOMŒOPATH.

F. E. Stoaks, M. D., Attica, Ohio.

For two years after graduating and assuming the responsibilities of a physician, and *pretending* to be a homœopath, the success which I enjoyed in curing disease was far short of what I anticipated. Cases presented themselves for treatment in due time after failure by allopathy to cure, and I, with that vain self-confidence which all young M. D.'s possess, more or less, promised them a sure cure. I did not completely fail, but it was so far from complete success that all the glory and hopes of victory and plenty of practice were converted into bitter disappointment. Some of my cases improved, others remained unaffected by my treatment, and none were cured, as I had so prematurely pictured in my mind. What was the matter? Was I or Homœopathy at fault? Was the science I had espoused, for which I had worked so hard to equip myself to represent, not what I had hoped it was? Was it a case of misplaced confidence? No. For I read in the journals of great cures, and the truth of *Similia* advocated, besides, my weak idea of what Homœopathy really was still lingered in "my hopeful breast." But I had not yet verified the "great truth" as I read it and had been taught. I had not seen a single really convincing result, from the cases I had treated thus far, to give me faith in the truth of *Similia.* I had some knowledge of materia medica which I had attained at college and in student life. Up to this time, dear reader, I had not read a single page of the *Organon.* Now, after this confession, you may imagine where the trouble was. I thought I knew what Homœopathy was, but I did not know as much about pure Hahnemannian Homœopathy as a two-year-old "kid" does of astronomy (excuse comparisons). I had not been under the instruction of the master. I had been groveling in the filth of materialism, routinism, empiricism, mongrelism, and everything but Homœopathy as Hahnemann practiced it. I had hitherto been guided by false ideas, or rather imperfect ideas, of the real principles and necessary requirements to be a successful homœopath. From reading a sample copy of THE HOMŒOPATHIC PHYSICIAN I was informed of the importance of studying the *Organon* in an article by the venerable Dr. P. P. Wells. I at once procured a copy and studied it, with the result of discovering how far I came from being a true homœopath, and how little I knew about Homœopathy.

After hesitating and reasoning for some time as to the truth of the dynamic force contained in drugs and the dynamic nature of diseases and the means of curing them by dynamic action as Hahnemann teaches us in Section 16, I resolved to treat my future cases according to Hahnemann. I had no medicine in my office higher than the 3x, except Lachesis 18th. I ordered a few of the polychrests in the thirtieth centesimal, and determined to treat my next case, whatever it was, with them, but not without considerable doubt of their power to cure. My first chance offered itself in the following case: A daughter came to my office for some medicine for her mother, who was "suffering with neuralgia." I explained that I must see her mother, which the girl requested me to do. I armed myself with a few of the thirtieth centesimal dilutions and went to my patient, whom I found in bed with her hands in her hair, uttering loud exclamations : " I shall die ! I can't stand it any longer !" *Tossing* about on the bed. Her face was red and expressive of great *anguish* and suffering. " *It seems as if my brain would burst out of my eyes.*" She would sit up a minute in bed, at which time I noticed her face would *turn pale*, and when she laid down it was *flushed.* Her skin was *hot* and *dry*, with *great thirst ;* pulse rapid and *thread-like.* I knew enough about materia medica to see Aconite strongly indicated, and therefore I gave it in the thirtieth centesimal, five drops in ten tablespoonfuls of water, and gave her one tablespoonful and waited. I was determind to follow Hahnemann in Sections 246–7. In five minutes my patient was noticeably easier, and in ten minutes was lying quietly sleeping, with a warm perspiration on her face. I would have you imagine my feelings at this moment. I promised to call next morning, when I found that my patient had slept all night. The medicine had not been repeated, according to orders, unless the pain returned. I was then realizing the truth of Hahnemann's teaching, and obtained more solid satisfaction by the result of this one case than in all the previous two years. We all need just such experiences as this to convince us of the truth of the efficacy of pure Homœopathy. This case was the means of arousing my anxiety to investigate further into Hahnemann's writings. I studied my *Materia Medica* and searched my repertories for the totality of symptoms. I am daily verifying the power of dynamic medicine, the single remedy and minimum dose, with the result of curing my cases easily and beautifully. I am now prescribing Dr. Swan's CM and CMM potencies with astonishing results. I care nothing about empty theorizing concerning the power of high potencies, but allow myself to be influenced only by results.

